# 10-degree reverse Trendelenburg position on hemodynamic parameters and block characteristics in unilateral spinal anesthesia in below knee orthopedic surgeries – can head up position do the trick?

**DOI:** 10.25122/jml-2022-0016

**Published:** 2022-10

**Authors:** Shyam Bhandari, Manuj Kumar, Aman Thakur, Sunil Thakur, Ravinder Kumar Verma, Bhanu Awasthi

**Affiliations:** 1 Department of Anesthesiology, Dr. RP Govt Medical College, Kangra, Himachal Pradesh, India; 2 Department of Anesthesiology, Dr. RK Govt Medical College, Hamirpur, Himachal Pradesh, India; 3 Department of Orthopedics, Dr. RP Govt Medical College, Kangra, Himachal Pradesh, India

**Keywords:** spinal anesthesia, Trendelenburg position, hemodynamic, orthopedic surgeries

## Abstract

Unilateral spinal anesthesia (USpA) is a technique used to restrict the effect of the spinal block on the operative side. 10–15 degrees reverse Trendelenburg position has been used to control the height of the spinal block using hyperbaric drugs. We aimed to study the effect of the 10-degree reverse Trendelenburg position on the quality of block and hemodynamic stability in unilateral spinal anesthesia in this hospital-based, double-blind, randomized clinical trial. 60 patients of both sexes between 20–60 years of age, undergoing below-knee orthopedic surgeries, were randomized into 2 groups. In both groups, spinal anesthesia was given with 2 mL bupivacaine heavy (0.5%), and the lateral position was maintained for 10 mins. Group 2 patients were kept in a 10-degree reverse Trendelenburg position throughout the surgery. The hemodynamic parameters and block characteristics of the two groups were compared using Epi Info statistical software. The onset of sensory block was faster in Group 1 (recumbent) compared to Group 2 (reverse Trendelenburg). The two-segment regression time was longer in the second group. In group 2, 73.3% of patients reached a level at T8 or below T8, compared to 46.7% in Group 1. The duration of sensory block and anesthesia was longer in Group 2. We conclude that reverse Trendelenburg of 10 degrees immediately after spinal anesthesia significantly limits the level of sensory block and prolongs the duration of unilateral spinal anesthesia.

## INTRODUCTION

Spinal anesthesia (SA) has long enjoyed a history of "anesthesia of choice" for below umbilical surgeries, being used by humanity for over a century [1]. Spinal anesthesia is often used for orthopedic surgery, especially in lower limb surgeries [2]. However, because of the high incidence of bradycardia and hypotension [3], there is always a risk, particularly in the elderly, given their compromised hemodynamic status [3, 4]. Unilateral spinal anesthesia (USpA) limits the spinal block level to the surgical side because most operative procedures involve only one lower limb [5]. Therefore, it produces fewer hemodynamic side effects and provides better stability, faster returns to daily routine activities, and better patient experience [5–8].

There are relatively few investigations in which the reverse Trendelenburg position was employed to control the height of the spinal block with hyperbaric medications [9, 10]. Furthermore, none of the studies used a head-up position and unilateral spinal anesthesia. Moreover, an extensive PubMed search could not show any studies comparing supine and head-up positions for unilateral SA for lower limb orthopedic surgery. Thus, we planned a study to evaluate the effects of the 10-degree reverse Trendelenburg position in unilateral spinal anesthesia, comparing it with a control group for block characteristics and hemodynamic parameters. We also attempted to examine whether using the reverse Trendelenburg position reduced the incidence of the most common spinal complications, namely hypotension and bradycardia, and whether this method was clinically beneficial.

## Material and Methods

This research was carried out in a "tertiary hospital" in northern India using a double-blind, randomized clinical trial including patients who underwent knee or sub-knee surgery. This analysis was carried out in line with the ethical standards of the 1975 Helsinki Declaration, as amended in 2000. Patients presenting for knee or below knee surgeries in a supine position under the subarachnoid block, aged 20–65, either sex, ASA physical status 1 or 2, and hemodynamically stable were involved in the research. Patients with ASA physical status of more than 2, any history of cardiovascular, renal, hepatic, respiratory, endocrine, and neuromuscular disorders, patients with epilepsy, neurological or psychiatric disorders, bleeding or coagulation disorders, local spinal deformity or local illness, and people with known allergy to local anesthetic agents were omitted from the research. Patients not consenting to regional anesthesia or patients who requiered surgery in a lateral position were also excluded from the study.

Randomization of participants was done using a computer-generated sequence with the help of sequentially numbered opaque envelopes to be opened by the concerned anesthetist. All observations were performed by the same observer to eliminate subjective errors. All participants gave informed consent in their native language during the preoperative visit. Ranitidine 150 mg and alprazolam 0.25 mg were administered on the night of surgery and 2 hours before surgery. The patient was transferred to the operating table, and a venous catheter was secured with an 18G cannula on the forearm. Loading was performed with 10 ml/kg of Ringer's lactate liquid. 5-lead ECG (electrocardiography), NIBP (non-invasive blood pressure monitoring), and SpO_2_ (oxygen saturation) were applied. Vital signs (SpO_2_, diastolic blood pressure, mean arterial pressure, pulse rate, and systolic blood pressure) were recorded before surgery.

An epidural catheter (Romson EPI KIT[GS-2006]) was placed into the L2-3 intervertebral space with an 18G Tuohy needle inside the lateral position using all aseptic precautions. The catheter was fixed after being inserted 4 cm into the epidural space. A subarachnoid puncture was performed at the patient's midline in a lateral position using a Quincke BD needle 26G×3.5-inch spinal needle that cuts the dura mater (0.45 mm×90 mm) in the L3-4 intervertebral space. Then, 10 mg heavy 0.5 percent bupivacaine was slowly inserted intrathecally at a 0.2 ml per sec rate. The beveled end of the spinal needle was kept facing down in the lateral position. There were 2 groups of patients. In Group 1, the patient stood in the lateral position for 10 min after spinal blockade without reverse Trendelenburg position and served as a control. In Group 2, patients stood in a lateral position for 10 min after the spinal block, with a reverse Trendelenburg of 10 degrees throughout the procedure. None of the patients was sedated, as this could interfere with our sensory blockade evaluation. Patients with incomplete block/partial effect or patients unable to perforate the L2-3 or L3-4 space due to epidural block and spinal block were excluded from the study. Blood pressure (mean, diastolic, and systolic), respiratory rate, heart rate, and SpO_2_ were monitored. Monitoring was started after completion of the injection, every 5 minutes until the first 60 minutes, and after that, every 10 mins until the surgery was over. Sensory analgesia was tested bilaterally along the midclavicular line every 30s until sensory blockage appeared (complete analgesia at T 12) and then every 5 min until both segments regressed below the maximal level.

A maximum degree of sensory block was seen 20 minutes after the intrathecal injection. The 2-segment regression interval was computed as the block regression time two segments down from the maximal sensory block. The sensory analgesia duration was determined as the time required for the block level to return to T12.

Requests for the first analgesic based on analgesia duration were noted. Motor blocks were tested using an adjusted Bromage scale [10]. Grade 0: No motor impairment; grade 1: inability to elevate extended leg; cable of moving knee and feet; grade 2: incapability to raise the extended leg and move knee; cable of moving feet; grade 3 included total motor block. The motor block was checked every 30 secs until it started and then each 5 min until the resolution of the block (withheld during surgery). The onset of motor block was calculated as the time taken between intrathecal injection to altered Bromage score 3, and the interval of motor block was considered as the time for motor block regression from adjusted Bromage scores 3 to 0. Adverse effects were noted, observed, and managed accordingly. Hypotension (systolic <90 mm Hg or mean blood pressure below 20% of baseline) was treated with Ephedrine 6mg intravenous stat, and in case of bradycardia (heart rate <45 beats/min), injection of Atropine 0.6mg intravenous was applied.

### Statistical analysis

GPower 3.1.9.7 was used to determine the sample size. Given a type I error of 5% and a study power of 80% to detect differences in one sensory segment, a sample size of 46 for two groups was calculated. Statistical analysis was conducted using Epi Info statistical software. Quantitative data were assessed with independent t-tests, and paired data were examined with paired t-tests. Qualitative data were assessed with chi-square and Fisher's exact test. A finding was deemed statistically significant if the value of p was below 0.05.

## Results

**Figure 1:**
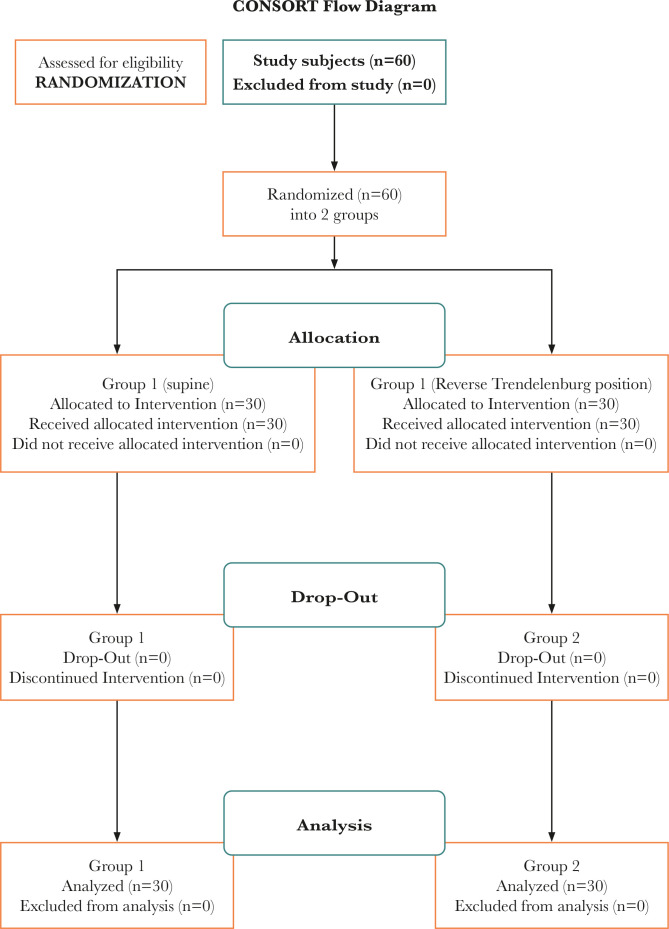
Flow chart of patients recruited and analyzed in two groups.

60 patients completed our study, as shown in Figure 1. Demographic parameters such as BMI (body mass index), weight, sex, height, age, ASA physical condition, and surgery duration were similar in the two groups (Table 1).

**Table 1:** Demographic profile of patients.

SN	Characteristics		Group 1 (Supine) (N=30)	Group2 (Head up) (N=30)	Statistical significance
**1**	Age (mean±SD)		38.7±12.73	42.4±11.71	P=0.2473
**2**	Weight (mean±SD)		59.7±5.71	58.6±5.28	P=0.4418
**3**	Height (mean±SD)		160±6.44	158.86±3.52	P=0.3984
**4**	BMI (mean±SD)		23.15±1.58	23.32±2.54	P=0.7573
**5**	ASA status	1	26	22	P=0.0173
	ASA status	2	4	8	

**Figure 2:**
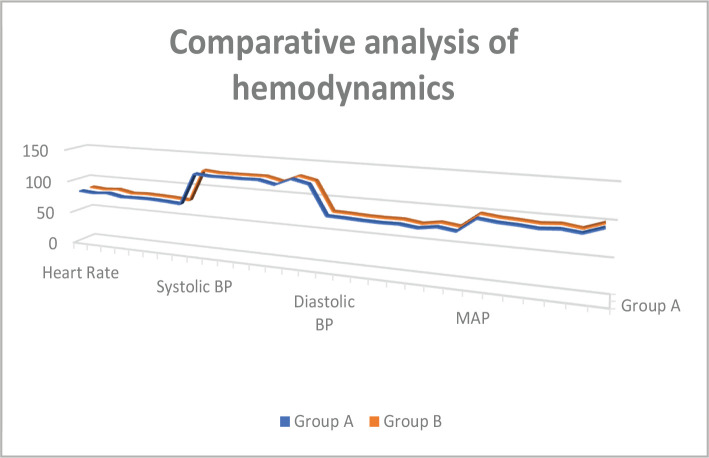
Line diagram showing comparative analysis of heart rate (HR), systolic blood pressure (SBP), diastolic blood pressure (DBP) and mean arterial pressure (MAP) among two groups.

The two groups were similar in intraoperative hemodynamic profiles, namely heart rate, mean arterial pressure (MAP), systolic blood pressure (SBP), and diastolic blood pressure (DBP) (Figure 2). The sensory and motor block features of the 2 groups are revealed in Table 2. The onset of sensory block was earlier in Group 1 (125.66±49.94 s) than in Group 2 (143±45.04 s). The onset of motor blockade was not substantially quicker in Group 2 than in Group 1.

**Table 2:** Comparison of block characteristics between the two groups.

S. NO	Variables	Group 1 (n=30) Supine	Group 2 (n=30) Head up	P-value
**1**.	**Onset of sensory block (seconds)**	125.66±49.94	143±45.04	P=0.1632
**2**.	**Maximum sensory block**			
	T4	1	0	P=0.0001
	T5	1	0	
	T6	13	4	
	T7	1	4	
	T8	1	4	
	T9	14	10	
**3**.	**No. of segments blocked above T8**	16	8	P≤0.05
	**No. of segments blocked at T8 or below T8**	14	22	
**4**.	**No. of segments**	5.13±1. 16	4.02±1.05	P=0.0001
**5**.	**Onset of motor block (seconds)**	297.66±62.7	279.66±63.43	P=0.2737
**6**.	**Two-segment regression time (min)**	72±7.94	86.16±12.50	P=0.0001
**7**.	**Duration of sensory block till T12 (min)**	123.33±17.6	144±18.21	P=0.0001
**8**.	**Duration of motor block (min)**	80.33±11.0	85.66±10.8	P=0.0648
**9**.	**Duration of analgesia (min)**	165.4±6.79	180.13±8.47	P=0.0001

**Figure 3:**
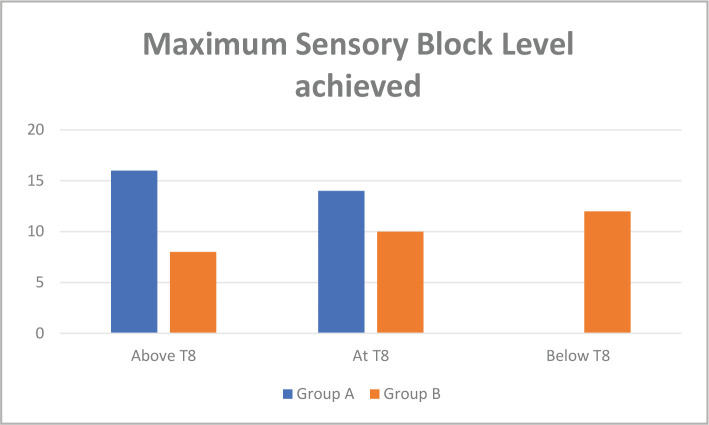
Bar diagram showing the level of the block above T8 and at T8 or below T8.

The maximum level of sensory block achieved was higher in Group 1 than in 2 (P=0.0001). The number of intermediate segments blocked at T12 or higher was higher in Group 1 than in Group 2 (p=0.0001). In Group 2, 73.3% of patients reached levels below T8 (or 26.7% of patients had sensory levels exceeding T8). In Group 1, 46.7% of patients reached levels below T8 (or 53.3% of patients with sensory levels above T8). Two-segment regression time was longer in Group 2 (86.16±12.50 min) than in Group 1 (72±7.94 min). The sensory block time was longer in Group 2 (144±18.21 minutes) than in Group 1 (123.33±17.6 min) (p=0.0001). The motor block time was longer within Group 2 (85.66±10.8 min) than in Group 1(80.33±11.0 min). Total pain relief duration was significantly longer in Group 2 (180.13±8.47 min) than in Group 1 (165.4±6.79) (P=0.0001) (Figure 3). In Group 2, there were no cases of severe hypotension compared to Group 1, in which 4 patients had episodes of hypotension requiring ephedrine. There was no episode of significant bradycardia in any of the groups.

## Discussion

Localized (unilateral) spinal analgesia in surgery was described in 1909 by Jonnesco [11]. Since that time, various techniques have evolved, each trying to confine the extent of the sensory and autonomic block to the site of operation. One of the most widely used techniques is unilateral spinal [12], in which limitation of spread is accomplished by using hyperbaric or hypobaric solutions, and segmental spinal [5], in which localization is affected by placing catheters to predetermined levels in the subarachnoid space.

The orthopedic anesthesia plan requires customization per the patient's need for a safe outcome. Low-dose bilateral and USpA with epidural anesthesia has advantages over conventional spinal anesthesia. It delivers more stable hemodynamics by providing intraoperative activation of epidural anesthesia and postoperative analgesia [5, 13]. The quest for regulating the height of the spinal block is as old as the history of spinal anesthesia. There has been a black period for spinal anesthesia during its discovery because of dreadful complications like hypotension, bradycardia, and postdural puncture headached (PDPH) [3, 11, 12]. The position of the patient might play a crucial role in assessing the final levels of motor and sensory blocks.

Low-dose spinal anesthesia and unilateral spinal anesthesia (USpA) with epidural anesthesia have advantages for orthopedic anesthesia. This ensures stable hemodynamics with a sufficient height of the spinal block. Spinal anesthesia has disadvantages such as hypotension and bradycardia because the block height cannot be predicted [3, 11]. The patient's position can play a role in examining the final level of motor and sensory blockade. An attempt was made to localize sensory blockade by maintaining patients in a supine position for 10–20 min [14, 15]. Hyperbaric bupivacaine was shown to prolong the period of pain relief [7, 16]. These results were confirmed by other researchers [5, 17–19].

In our work, the onset of sensory blockade was quicker in Group 1 than 2. Ciceki et al. [20] found similar results. In this study, the peak block level remained confined to the T8 level in most patients in Group 2, resulting in a denser sensory blockade compared to Group 1. The findings were statistically significant (P=0.0001). Similar findings were obtained by Magar et al. while studying unilateral spinal anesthesia [21]. This study found that the reverse Trendelenburg position limited the number of spinal segments blocked. This control in the level of spinal block may be of great help in geriatric and high-risk patients where a high level of sympathectomy can be detrimental due to increased hypotension and bradycardia.

The analgesia duration was also substantially prolonged in the reverse Trendelenburg group. This research was assisted by a similar work [21]. Group 2 did not require vasopressors, whereas 4 patients in Group 1 required vasopressors for hypotension. Stable hemodynamics in the reverse Trendelenburg group have been reported in earlier studies [22].

The difference in regression time found in our study was only 10–15 min. but was similar to that of Lee et al. [9]. Our conclusion was also confirmed in another study by Borghi B et al. [23] with a two-segment regression time of 96±3.2 min. In our study, the reverse Trendelenburg posture significantly increased the duration of sensory blockade. Similar results were obtained by Magar et al. [21]. However, no study used a head-up position in spinal anesthesia for lower extremity surgery.

One of the important limitations of our study is the use of 10 mg of the drug, which may be too high to produce a true hemispinal. Also, results of the reverse Trendelenburg position cannot be extrapolated to other population groups like obstetric patients due to physiological changes in pregnancy. Further larger sample-sized clinical examinations are needed to confirm our hypothesis.

## Conclusion

We conclude that keeping patients in reverse Trendelenburg of 10 degrees immediately after providing spinal anesthesia considerably reduces the level of sensory block. It can be clinically advantageous in controlling block height in high-risk patients involving lower limb surgeries where levels above T8 are rarely required. At the same time, low sensory block levels led to prolonged analgesia duration. Therefore, we recommend further studies using the reverse Trendelenburg position in geriatric and other high-risk groups. It can be a ray of hope to find a way that has not been achieved perfectly until today since the inception of spinal anesthesia a century ago.

## Data Availability

Further data is available from the corresponding author upon reasonable request.
